# Common Mechanisms of Long COVID and Vascular Dementia

**DOI:** 10.1002/alz.089568

**Published:** 2025-01-09

**Authors:** Grant Talkington, Saifudeen Ismael, Gregory W Hall, Gregory J Bix

**Affiliations:** ^1^ Tulane University, New Orleans, LA USA

## Abstract

**Background:**

Vascular dementia (VaD), the second most common cause of dementia, is characterized by cognitive decline due to reduced cerebral blood flow and blood‐brain barrier disruption. Current evidence demonstrates that not only are VaD patients at higher risk of severe COVID‐19 illness and mortality, but also that pre‐existing cognitive dysfunction/dementia is associated with increased COVID‐19 incidence. Conversely, SARS‐CoV‐2 infection alone worsens dementia‐related mild cognitive impairment (MCI) and increases risk of cognitive decline, supported by similar fMRI findings demonstrating hypoperfusion. Therefore, we hypothesize that both vascular dementia and long COVID bear a common vascular mechanism.

**Method:**

To investigate, we conducted a chronic‐phase COVID‐19 study utilizing an innovative non‐transgenic mouse model with Mouse Adapted 10 (MA‐10) strain of SARS‐CoV‐2 following bilateral carotid artery stenosis (BCAS) to model VaD. We inoculated 12‐week‐old C57 mice intranasally with 1×10^4 PFU of MA‐10 and followed them to 15 days post‐infection.

**Result:**

Bulk RNA sequencing on brain tissues and analysis with Ingenuity Pathway Analysis (IPA) revealed substantial net alterations to several signaling pathways including IL‐6, IL‐1B, and Pathogen‐associated molecular response patterns, providing critical insights into the mechanisms underpinning acute and chronic COVID‐19 morbidities.

**Conclusion:**

Our findings confirm a significant mechanistic interaction between VaD and long COVID, suggesting that existing therapies targeting VaD may also be effective in long COVID.

